# Plants used during pregnancy, childbirth and postpartum healthcare in Lao PDR: A comparative study of the Brou, Saek and Kry ethnic groups

**DOI:** 10.1186/1746-4269-5-25

**Published:** 2009-09-08

**Authors:** Hugo de Boer, Vichith Lamxay

**Affiliations:** 1 Department of Systematic Biology, Evolutionary Biology Centre, Uppsala University, Norbyvagen 18D, SE-75236 Uppsala, Sweden; 2 Department of Integrative Biology, University of California, Berkeley, 3060 Valley Life Sciences Bldg #3140, Berkeley CA 94720, USA; 3 Department of Biology, Faculty of Sciences, National University of Laos, Dongdok campus, Vientiane, Lao PDR

## Abstract

**Background:**

In many Southeast Asian cultures the activities and diet during the postpartum period are culturally dictated and a period of confinement is observed. Plants play an important role in recovery during the postpartum period in diet, traditional medicine, steam bath and mother roasting (where mother and child placed on a bed above a brazier with charcoal embers on which aromatic plants are laid). This research focuses on the use of plants during pregnancy, parturition, postpartum recovery and infant healthcare among three ethnic groups, the Brou, Saek and Kry. It aims to identify culturally important traditions that may facilitate implementation of culturally appropriate healthcare.

**Methods:**

Data were collected in 10 different villages in Khammouane province, Lao PDR, through group and individual interviews with women by female interviewers.

**Results:**

A total of 55 different plant species are used in women's healthcare, of which over 90% are used in postpartum recovery. Consensus Analysis rejects the hypothesis that the three ethnic groups belong to a single culture for postpartum plant use, and multidimensional scaling reveals non-overlapping clusters per ethnic group.

**Conclusion:**

Medicinal plant use is common among the Brou, Saek and Kry to facilitate childbirth, alleviate menstruation problems, assist recovery after miscarriage, mitigate postpartum haemorrhage, aid postpartum recovery, and for use in infant care. The wealth of novel insights into plant use and preparation will help to understand culturally important practices such as confinement, dietary restrictions, mother roasting and herbal steam baths and their incorporation into modern healthcare.

## Background

Medicinal plants have a significant role during pregnancy, birth and postpartum care in many rural areas of the world. Plants used in women's health related conditions such as female fertility, menorrhea, birth control, pregnancy, birth (parturition), postpartum (puerperium) and lactation, including infant care, have been documented for various ethnic groups (e.g. [1-6]). In Western traditional medicine the label "old wives' tales" has been applied to all knowledge of interest to women - fertility, birth, childcare - transmitted orally from one generation of women to the next, and the derogatory label reflects male devaluation and relegation to folklore of this exclusively female realm of knowledge [7]. Research focusing on the use of these plants often focuses on the realm of knowledge of male traditional healers, and scholars have missed the wealth of knowledge that is held by women [8]. It has been suggested that this expertise being exchanged and applied in secrecy as traditional knowledge relating to women's health, empowers women and undermines male dominance [7]. Pregnancy, parturition and the puerperium each mark a significant step in *matrescence *[9], and are not without risk to the mother and infant. According to the latest data, the infant mortality rate (deaths per 1000 live births) and maternal mortality (maternal deaths per 100 000 live births) for Laos is 60.3 and 660, respectively [10,11]. By comparison, those numbers for Sweden are 3.2 and 3 [10,11].

In the context of the introduction and modernization of primary healthcare systems in rural areas, and with training programs for traditional birth attendants focusing on the paradigms of Western medicine, this traditional knowledge has often been ignored [12]. Erosion and deterioration of traditional medical knowledge can be observed in many cultures and leads not only to a loss in biocultural diversity, but also diversity in alternatives for primary healthcare [13]. Documenting the use of plants by ethnic minorities is not only an important part in understanding and analyzing elements of traditional birth practices, but a way to perpetuate knowledge at risk of being lost.

The postpartum period is important in many Southeast Asian cultures, and is seen as a period of recovery and often entails a period of confinement ranging from 10 up to 45 days. In accordance with humoral medicine pregnancy is seen as a hot state; with parturition heat is lost and the woman comes into a state of excess cold, and during the postpartum period care should be taken to restore the mother to equilibrium [14]. Confinement as a treatment involves staying inside and near heat, washing only with hot water, drinking hot drinks, eating hot food, and staying away from draughts [14]. Confinement as a term is fairly broad and can include steam bath and bathing, mother roasting, dietary proscriptions and consumption of medicinal plant decoctions.

Steam bath is common throughout Southeast Asia (for review see [14]. Steam baths or baths often involve making a decoction out of medicinal plants. The participant may either take a bath directly as is common for the Yao in Laos, Thailand and Yunnan province, China [15], sit in a special room into which steam from the decoction is led [14], or sit in a tent-like construction with [3], or on a seat-less chair over [6], a pot containing the steaming decoction. By lifting the lid of the pot the steam is let out, containing essential oils and other volatile substances, for inhalation and absorption through the skin, and as the water cools down the mother uses the decoction to cleanse the body.

Mother roasting is culturally related to steam bath, but differs in that the convalescent individual lies on a bed placed above a brazier with charcoal embers on which aromatic plants are laid, thus enabling the essential oils to vaporize. Mother roasting is often done to 'dry out' and to stop the lochia, restore the uterus to pre-pregnancy condition and to alleviate postpartum abdominal pain [14]. Mother roasting seems to be confined to Southeast Asia, where it is widespread and reported from Indonesia [16], Laos [17,18], Malaysia [19], Myanmar [20], the Philippines [21], Thailand [22] and Vietnam [23]. Treatments similar to mother roasting have been documented amongst native Americans from Pacific North America [24], but here the mother lies on a bed covered in plants that are *steamed *from beneath. Van der Eerden [25] describes a treatment amongst Spanish-Americans in New Mexico more similar to mother roasting, where postpartum haemorrhage is treated by spreading lavender over burning coals and having the mother stand in the smoke. Little is known about the plant species used in mother roasting, but the use of the leaves of turmeric (*Curcuma longa *L.) to prevent the coals from burning is reported from Thailand [22].

Diet is important during pregnancy, confinement and in some cultures also after confinement [9]. Postpartum avoidance of foods classified as cold, such as fresh fruits and vegetables, cold foods, and plain water, is almost universal. Many ethnic groups report the prescription of hot decoctions of ginger (*Zingiber officinale *Roscoe) or turmeric (*Alpinia galanga *(L.) Sw.), boiled rice, boiled vegetables and boiled chicken or fish, all combined with salt for drying out the womb [14].

This study focused on all plant use during pregnancy, parturition, and postpartum for lactation and postpartum recovery among three poorly studied ethnic groups, the Brou, Saek and Kry, in Khammouane province, Lao People's Democratic Republic. All three groups practice subsistence agriculture, are animists practicing ancestor-worship and live in wooden houses raised on stilts. All three groups are ethno-linguistically more closely related to groups living in other areas. The Kry are hypothesized to have originated from this area [26], whereas the Saek would originate just across the Vietnamese border [27]. The Brou form part of larger group and are thought to have arrived in the area later from Quang Tri province, Vietnam [26].

The Brou have an estimated population in Laos of 69,000 [28], and inhabit the Annamite mountains in Khammouane and Savannakhet provinces, as well as the adjacent area in Vietnam. They speak a Katuic language in the Mon-Khmer subgroup of Austroasiatic, are animists, live in raised houses on wooden stilts and practice swidden agriculture as well as some paddy rice cultivation [26]. Brou culture has not been thoroughly studied. For the Makong (which is an officially used group including the Brou [26,29]), it is reported that pregnant women are supposed to work hard and sleep little, as activity during pregnancy will make for an easy childbirth. During pregnancy the father is proscribed killing animals as this may cause deformity in the child; and the mother should avoid eating bananas, beef or pork. Birth is reported to take place in special birthing huts, placed near the house, where the mother will stay up to 3 days after giving birth, and religious rites are performed before she is allowed to return home. Postpartum diet for the first three days consists solely of rice and salt. [29]

The Saek live mainly in the Bolikhamxay and Khammouane provinces in Laos, with some smaller communities in Nakhon Phanom province in Northeastern Thailand, and their language belongs to the Tai-Kadai language group. Population estimates for them vary from 2,700 [29] to 25,000 [27]. Nothing is known of their practices with regard to pregnancy, childbirth and labor.

The Kry consist of about 230 people living in a few settlements in the Nam Noi watershed, Khammouane province [30], speaking a Vietic language in the Mon-Khmer subgroup of Austroasiatic. The Kry are animists and have traditionally lived a nomadic live-style in small bands as hunter-gatherers. Settlement in and near villages of Lao and other ethnic groups in recent decades has led to cultural amalgamation, and today few communities exist with a majority Kry population [26]. Nothing is known of their practices with regard to pregnancy, childbirth and labor.

The data presented in this study provides a first insight into medicinal plant species used in women's healthcare by the Brou, Saek, and Kry ethnic groups in Laos, and assesses using a quantitative approach whether postpartum plant use is shared among the ethnic groups as one culture, and if patterns of postpartum plant use can be identified. The study aims to identify culturally important traditions surrounding pregnancy, childbirth and postpartum recovery that may aid in the development and implementation of culturally appropriate healthcare by the Lao government or non-governmental organization working in this field.

## Materials and methods

### Study site

The data presented here were collected in December 2005 - February 2007 in 10 villages in the Annamite Mountains in the Nakai-Nam Theun National Biodiversity Conservation Area, Nakai District, Khammouane Province, Lao People's Democratic Republic: 1 on the Nakai Plateau; 1 in the Nam Theun valley; 4 in the Nam Noi valley; and 4 in the Nam Pheo valley, all situated above the Nakai Plateau (Table 1).

**Table 1:** Villages included in this study with background information

**Village**	**Locality^1^**	**Alt. (m)**	**Ethnic group^2^**	**# of households**
Ka Oy	17°43.70' N 105°20.12' E	529	Brou/Lao	32

Mak Feuang	17°50.45' N 105°21.52' E	555	Brou	62

Phung	17°50.69' N 105°27.31' E	602	Brou	24

Koutnae	17°50.91'N 105°34.57' E	601	Brou	22

Makaa Tai	17°55.27' N 105°30.37' E	622	Kry	18

Makaa Kang	17°55.91' N 105°31.73' E	631	Kry	21

Dteung	17°54.10' N 105°29.00' E	607	Saek	55

Buuk	17°51.16' N 105°32.52' E	588	Saek	28

Nameo	17°50.79' N 105°32.87' E	597	Saek	30

Nameuy	17°50.82' N 105°33.29' E	589	Saek	62

^1 ^Coordinates are given in degrees, minutes and decimal minutes; ^2 ^Due to transliteration, as well as alternative interpretations, multiple spellings and names exist for most Lao ethnic groups: 1) **Brou**, Brue, Makong; 2) **Kry**, Kree, Kri, Salang, Makaa, Labree, Yubree, Arehm; 3) **Saek**, Xaek, Xe'k, Sek.

### Interviews

A total of 38 interviews and 12 plant collecting walks were conducted in 10 villages, belonging to 3 ethnic groups: the Saek, Brou, and Kry. Note that various different transliterations exist for the names of these ethnic groups: 1) *Saek*, Xaek, Xe'k, Sek; 2) *Brou*, Brue, Makong; 3) *Kry*, Kree, Kri, Salang, Makaa, Labree, Yubree, Arehm. Interviews were carried out in the periods December 2005, June 2006 and February 2007. All villages were geo-referenced using a Garmin E-trex Venture GPS receiver (Table 1). All interviews were conducted in Lao.

Data for the villages Buuk, Dteun, Koutnae, Makaa Kang, Makaa Tai, Mak Feuang, Nameo, Nameuy and Phung were collected in June 2006 and February 2007, and data for the villages Ka Oy and Mak Feuang were collected in December 2005. Data collection was done using the following general format: interviews were conducted in the homestead. After introducing the research team and research objectives to the head of the village, an informal open-ended interview was conducted to collect demographic and social data about the village. Subsequently two group interviews were conducted: one with male informants selected by the head of the village as people knowledgeable on plant use; and another by female interviewers with female informants: the village midwife, nurse and other knowledgeable women. Group interviews were culturally readily acceptable, but valuable data may be overlooked, as verbal dominance may not correlate with traditional knowledge. Both interviews used free listing of medicinal plants per usage class to elicit information. The male interviews consisted of classes of ailments, and in addition plants used during pregnancy, childbirth and labor. The female interviews focused on pregnancy, childbirth and labor, and in addition plants used in women's healthcare and for treating diseases in children. Following the interviews walks were made in the surrounding forest to collect the plants mentioned during the interviews by male, female or mixed teams. Additional information was recorded while pressing the plants for herbarium vouchers.

### Botanical collections

Plant names mentioned during the interviews were recorded in Lao and transliterated from the ethnic group's language to Lao script or Roman script using French phonetics as is common in Laos. Plant material was collected, pressed and drenched in alcohol for herbarium vouchers and subsequent identification. A complete set of herbarium vouchers was deposited at the herbarium of the Department of Biology of the National University of Laos and at the Uppsala University Herbarium (UPS). Common cultivated species were identified in the field, using the local name, and/or a checklist of Lao and scientific names [31].

### Data analysis

Expected species accumulation curves of the number of species reported on postpartum plant use were computed using the Sobs (Mao Tau) function [32] in EstimateS 8.0 [33], which is a randomized resampling-based method, to assess the coverage of the sampling.

The reported number of species used in postpartum healthcare per village was analyzed in Anthropac 4.98 [34] to test for consensus between the villages. Consensus analysis is a technique for discovering patterns of agreement and disagreement concerning a domain of knowledge among individuals or groups [35]. Data was dichotomized, tested for similarity using positive matches and plotted using non-metric multidimensional scaling [36] following Puri & Vogl [37]. Data was further analyzed using the property fitting module PROFIT, and quadratic assignment procedure module QAP regression, in Anthropac 4.98 [37,38]. Dependent variables were the presence-absence adjacency matrix of plant species used in postpartum healthcare mentioned per village. The attribute matrix consisted of categorical data on ethnicity (Brou, Saek, or Kry) as well as quantitative data on proximity of village to next Brou, Saek, Kry or village of the same ethnic group (in km). Distance was approximated using the shortest distance between two villages, located using GPS coordinates taken in the field, in the GoogleEarth mapping system. Categorical attribute variables were independently converted to matrices by scoring for matches in a single-variable similarity matrix [37].

## Results and Discussion

### Plants used in women's health

**Figure 1:**
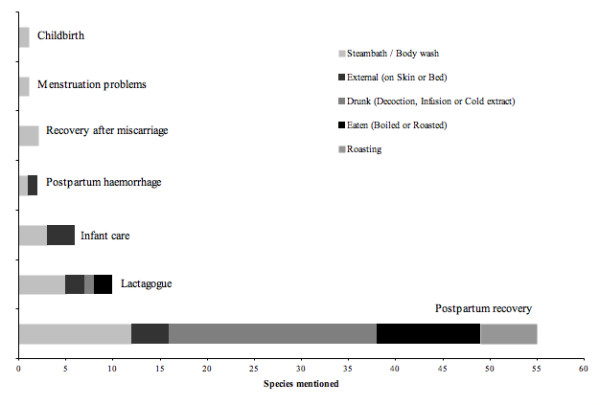
**Bar diagrams of plant species mentioned for use in women's health per category, showing absolute values, subdivided by the reported type of use**.

Plant use during pregnancy, childbirth and postpartum healthcare among the Saek, Brou and Kry ethnic groups inhabiting the upper Nam Theun, Nam Noi and Nam Pheo is common with 55 species mentioned during 38 interviews. A summary of all respondent data (Additional File 1) allows for a quick overview of plant species used, part used, treatment and preparation per village and ethnic group. Plants were reported to be used during childbirth, to treat menstruation problems, for infant care and postpartum recovery. The latter could be subdivided into recovery after miscarriage, postpartum haemorrhage (locally defined as unusually profound bleeding), lactagogue and normal postpartum recovery. The reported uses are classified into the following application categories: Steam bath and body wash; External use either applied as poultice or placed on bed or sleeping mat; Oral use, as decoction, infusion or cold extract; Eaten, either boiled or roasted; and used for Mother Roasting (Figure 1).

Twenty-six of the 55 species are used in steam bath and mother roasting, which are both methods in which the plant material is heated, allowing for the release of essential oils through evaporation, with the intention to benefit from the medicinal properties of these oils through inhalation. Inhalation and transdermal application of essential oils has documented pharmacological effects [39]; and plants used in these treatments are likely to contain high levels of volatile compounds. Nine of the 26 species documented in this study belong to the plant families, Asteraceae, Lauraceae, Rutaceae, or Zingiberaceae, all of which are known for their essential oil content [40]. Five of the reported species belong to the family Poaceae, which has only few aromatic species, and the use of these species possibly serves other purposes, e.g. slowing the release of volatiles during boiling through chemical binding or attraction, or simply by increasing the mass of added plant material to the boiling water or on the glowing embers.

### Plant source

In Brou, Saek and Kry society both men and women work in the rice fields and collect non-timber forest products along the way between the often distant forest swiddens, but men unlike the women also venture into the primary forest for hunting. All 55 plant species used in women's healthcare identified in this study are harvested from secondary forest or disturbed vegetation, which is consistent with the disturbance pharmacopoeias theory [41] of the importance of plants from secondary vegetation in traditional medicine, and also the importance of secondary vegetation for medicinal plant collection by women due to gender-based differences in what areas men and women visit [8].

### Scientific literature on plant species

Most of the medicinal plants in this study can be found in scientific literature reported for the same or similar affections. Spire [17] reports that *Globba calophylla *Ridl. is taken postpartum as a decoction in Xieng Khouang province in Laos. Manderson [19] reports that *Blumea balsamifera *(L.) DC. and *Ageratum conyzoides *L. are used in postpartum recovery in Peninsular Malaysia. Wang et al. [3] report the postpartum use of *Ageratum conyzoides *L., *Bischofia javanica *Blume, *B. balsamifera*, *Chromolaena odorata *(L.) R.M. King & H. Rob. by the Haw in Northern Thailand. Anderson [42] in his book on ethnobotany of the Akha, Hmong, Lahu, Lisu and Mien (Yao) reports all but 10 species found in this study. Eight of these ten are cultivated species that are mainly used as food and lie outside the focus of that study, and the remaining two are *Eurycoma longifolia *Jack and *Rhapis laosensis *Becc. The root of *E. longifolia *is reported to be used as a febrifuge, expectorant, to relieve gastric pains, reduce high blood pressure, and used as a poultice on wounds, ulcers and sores, the leaves to relieve stomach-ache, and the bark as a blood coagulant in complications during childbirth [43]. No literature exists on the use of *R. laosensis *or species in the genus *Rhapis*. However, young palm roots of the genera *Caryota *and *Arenga*, which are similar in appearance to *Rhapis*, but known for their ethnobotanical use, were mentioned as being used by informants in this study for other medicinal uses. Both *Rhapis*, *Caryota *and *Arenga *were identified as *tao*, and this possibly reflects an under-differentiation in the local ethnoclassifications of young sterile palms.

### Data analysis

**Figure 2:**
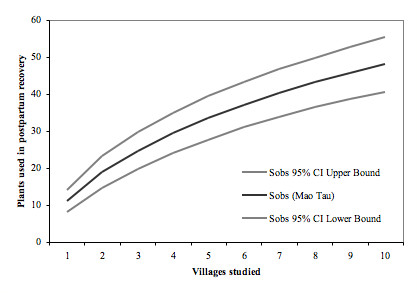
**Expected species accumulation curve computed using the Sobs (Mao Tau) function (Colwell et al. 2004) in EstimateS 8.0 (Colwell, 2006) to assess exhaustiveness of the collected data on plants used in postpartum care**.

The expected species accumulation curves computed in EstimateS 8.0 (Figure 2) level off as the number of interviews increases, indicating a reduction in the number of new species mentioned per interview. This is consistent with the underlying hypothesis that a limited number of species are used and that the most salient species can be elicited after a limited number of in-depth interviews. The slope of the curve after interviewing in 10 villages is still significant, and further interviews are likely to elicit additional less salient species.

**Figure 3:**
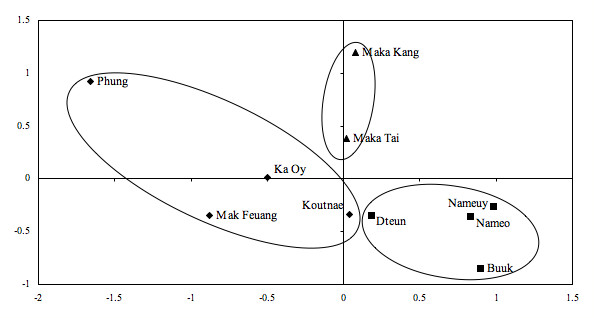
**Similarity in postpartum plant use plotted using non-metric multidimensional scaling (Kruskal Stress 0.048)**. Distances between points are relative similarity. Diamonds represent villages belonging to the Brou ethnic group, squares are Saek and triangles are Kry. Consensus Analysis rejects the hypothesis of one culture for postpartum plant use (eigenvalue ratio 1.816; pseudo-reliability 0.844), but clustering of ethnic groups is evident.

Consensus Analysis of postpartum plant use between the villages measured as plant species used in postpartum recovery per village reject the one culture hypothesis of plant use for all ethnic groups (ratio of 1^st ^to 2^nd ^eigenvalue is 4.053/2.232 = 1.816, pseudo-reliability 0.844). Plotting of the consensus analysis similarity matrix using non-metric multidimensional scaling (MDS) in two dimensions has a Kruskal Stress of 0.048 [36] and is an accurate representation of the similarity in knowledge between the villages, and each ethnic group forms a separate cluster (Figure 3). The coordinates in the MDS plot are based on similarities in postpartum plant use, and allow for comparison of villages and ethnic groups. Plant use is similar in all villages (Additional File 1), but arbitrary clusters can be drawn around each ethnic group (Figure 3).

Computations in Anthropac 4.98 of matrix regression using the quadratic assignment procedure of the categorical variable ethnic group, and property fitting of quantitative independent variables of distances between nearest village and distances to nearest village of the same ethnic group gave no significant results. The limited distances between the clusters relative to the internal variation likely explains the lack of statistical support for the observed clusters.

### Childbirth traditions

Childbirth and confinement take place at home for Brou and Saek mothers, and usually only the husband and one or more midwives are present during labor and parturition. The husband and midwife give both mental and physical support during the entire process. In case of complications midwives or knowledgeable women from the village, other villages or nearest healthcare facility may be called in for help. Getting help from outside the village can be time consuming as the main mode of transport is by foot.

The Kry give birth outside the village in a simple shelter constructed by the village for the purpose of giving birth, and this tradition probably reflects the practical constraints of a former small-band nomadic life in the forest, where privacy would be limited in a makeshift camp. At the onset of labor the woman leaves the village with a midwife and possibly her husband and goes to the shelter, which is located downstream of the village near the river. Mother and child need to stay there for 3-5 days after birth, and during this time she only consumes salt water (half a tablespoon of salt mixed with 3 tablespoons of warm boiled water). Subsequently she and the infant move to a special hut in the village, either attached to the main house or built close to it, for another 10-15 days of recovery. Kry women stay in these specially constructed huts during their menstrual period and the second stage of convalescence. In addition they may not use the regular places in the river for bathing, but must go either further downstream or elsewhere.

Giving birth in the forest is also reported for the Katang in Laos. Traditionally Katang women in labor leave the village and give birth in the forest supported by their husband and a traditional midwife. A small camp with a fire is set up for use during parturition and the first few days postpartum, however during the rainy season a makeshift hut may be constructed for shelter [44].

#### Plant use during pregnancy and childbirth

Ninety-one percent of the total number of species mentioned was reported to be used in postpartum recovery, and plant use during pregnancy and parturition appears to be less common. The limited number of plants recorded for use during pregnancy and parturition is supported by interviews with the informants. The people in Dteun report specifically that no plants are used during childbirth, in Nameo and Nameuy no traditional medicine is used during pregnancy, and in Ka Oy and Mak Feuang herbal medicine, alcohol and modern medications are avoided during pregnancy. Whether these restrictions are based on traditional perceptions of teratogenicity or abortive properties would be interesting to study. In general in Southeast Asia, pain is seen as a natural part of childbirth, and is alleviated only by massage, reassurance and emotional support [45].

#### Confinement

It is common among all ethnic groups in this study to observe a period of confinement after childbirth, combined a special diet, steam baths and washing with herbal decoctions, consumption of herbal decoctions and mother roasting. Confinement is reported to be approximately one lunar month for the first child; and less for subsequent children, ranging from 10-15 days. The people in Mak Feuang and Ka Oy, who are nearest the town of Nakai, stated that confinement was observed less strictly nowadays due to food shortage and the increased workload, but that first time mothers would often stay in confinement for a full lunar month. Reduced or relaxed confinement is common in industrialized areas in Southeast Asia [14].

#### Postpartum diet

The postpartum diet consists of food dietary prescriptions and proscriptions, as well as the consumption of herbal decoction, infusions and cold extracts. Informants in Phung report that a diet of rice, salt and galanga (*Alpinia galanga *(L.) Sw.) is eaten after birth for recovery. Saek informants in Nameo and Nameuy stress the importance of a confinement diet of hot water, combined with boiled rice with boiled vegetables, boiled unripe banana flowers, boiled rattan shoots and salt, chicken or fish soup. Brou informants in Mak Feuang and Ka Oy observe a confinement diet of boiled rice, vegetables, fish and salt. Chicken, preferably with dark meat, is eaten first after 15 days of confinement. Cold foods, uncooked water, fresh vegetables and fruit were avoided.

Consumption of decoctions, infusion and cold extracts was common in all villages and among all ethnic groups. Specific plant extracts were drunk to facilitate childbirth, alleviate menstruation problems, assist recovery after miscarriage, mitigate postpartum haemorrhage, aid postpartum recovery, or for use in infant care. Plant extracts used for postpartum recovery were cited as helping to expel the lochia, lessen mild postpartum haemorrhage, contract the uterus, and give the mother strength.

#### Mother Roasting

The effects and pharmacology of the process of mother roasting, where the mother is placed on a bed over a open heat source with aromatic medicinal plants, such as a charcoal brazier, deserves further investigation, and seems to be confined to the Southeast Asia region [14]. Mother roasting is an integral part of postpartum recovery and confinement, and the reported purposes are to dry the womb, stop the lochia, restore the uterus to pre-pregnancy condition, and alleviate postpartum abdominal pain. The practice of mother roasting is reported for all villages, and is done in similar fashion to the description above. It is important to bear in mind that people sleep on mats or mattresses on the floor, and that the bed used for mother roasting is a purpose-built bed. The heat source underneath the bed is not a brazier, but glowing embers on a laterite platform similar to that used in the kitchen.

Mother roasting periods vary per village, and Saek informants in Nameo report that confinement and roasting is done for 30 days after the birth of the first child; but for only 10 days after subsequent births. Roasting can start immediately after childbirth, when the child is placed on the bed with the mother. In Nameuy roasting is done for one lunar month following the birth of the first child, and for later children 7-10 days. In Buuk roasting was reported for a period of 20 days after birth of the first child; and 5-6 days for subsequent children if the mother is healthy, but 10 days if she is not.

#### Steam bath and washing

Herbal steam bath and washing with herbal decoctions is common during confinement throughout Southeast Asia, and is practiced in all villages in the study. The purpose of the steam bath is to cleanse and heat the body, and benefit from the medicinal properties of the herbs used. The Brou in Ka Oy and Mak Feuang describe their practice of steam bath as follows: The individual exposed to the treatment sits under a thick blanket on the floor or bed with a pot of boiled water containing medicinal plants. By lifting the lid of the pot she lets out the steam, containing essential oils and other volatile substances, for inhalation and absorption through the skin, and as the water cools down she uses the decoction to wash her entire body. Frequency and duration of steam bath varies between ethnic groups, villages and for first or subsequent childbirths. Informants in Dteun report the use of steam bath for postpartum recovery during the first 10 days after childbirth, 3 times per day and 3 times per night, as well as washing with a decoction of medicinal plants 3-4 times per day, which is similar to the use in Buuk, where steam bath and washing are done for 20 days after the first child's birth, and 10 days after subsequent children's births, for 3 to 4 times per day. Commercial steam baths in Vientiane and Luang Namtha report similar time spans for postpartum recovery treatment, but recommend use only twice daily and to start between 7-10 days after parturition [46], possibly for practical and hygienic reasons.

## Conclusion

Plant use is common during postpartum recovery among the Brou, Kry and Saek ethnic groups. Observing a period of confinement for the mother and newborn infant is common during which a variety of treatments are practiced, such as mother roasting, steam baths, cleansing with herbal decoctions, drinking herbal decoctions and infusions and abiding by food proscriptions and prescriptions. These treatments and the plant species used in the treatments aim to relieve postpartum abdominal pain, reduce postpartum haemorrhage, aid in physical recovery, augment lactation, and treat illness in infants. Knowledge of the plant species used, where to collect them, how to harvest them, how to prepare them and how to use them is an important realm of knowledge possessed by women in these communities, but shared with men.

These traditions are common and widespread in Southeast Asia, and form the core of primary maternity healthcare in many rural areas in Laos. Modernization of healthcare in Laos could benefit from incorporating these treatments and their plant use into healthcare modernization programmes through active involvement of traditional rural midwives. It would facilitate the implementation of culturally appropriate healthcare that respects traditional knowledge and contributes to bio-culturally sustainable development.

Increasing the existing documentation of ethnobotany of the ethnic groups inhabiting mainland Southeast Asia: Laos, Northern and Northeastern Thailand, Vietnam, Myanmar, Cambodia and Yunnan Province, China is essential as rapid assimilation with mainstream culture increases. Data collection aimed specifically at plant species used in women's healthcare remains scarce, and general ethnobotanical studies often overlook the variety and relative importance of plants used in women's healthcare. Recent ethnobotanical studies in the area report few species used in women's healthcare [47-51]. Anderson [42,52,53] studied the Lisu, Lahu, Karen, Akha, Mien (Yao) and Hmong extensively in Northern Thailand and identified nearly 700 species of medicinally used plants, of which at least 115 are used in women's healthcare. Weckerle et al. [51] studied the Bai in the Shaxi Valley, Yunnan and list 176 medicinal plant species of which 16 can be classified as used in women's healthcare. The research by Anderson and Weckerle did not focus specifically on women's health and illustrates that a meticulous study, regardless of a gender bias, can nonetheless document medicinal plant use in women's healthcare.

The rich cultural diversity of the ethnic groups in Southeast Asia is emphasized by the divergence in knowledge found in this study of three ethnic groups inhabiting the same geographical area. There is a clear need for ethnobotanical research to document plant use, and in particular to focus on traditionally ignored subjects such as women's health care. Acknowledging limitations in breath of results in publications focusing solely on the male knowledge realm is another aspiration. Research focusing on the pharmacological mechanisms and the efficacy of these treatments, that are both ancient and widespread, could provide insights that could help to augment and improve both local and Western postpartum care.

## Competing interests

The authors declare that they have no competing interests.

## Authors' contributions

HdB and VL conceived the research. VL was responsible for field research and interviews. HdB and VL collected all the data in the field and identified the herbarium vouchers. HdB processed the data, performed the quantitative analysis and drafted the manuscript. All authors read and approved the final manuscript.

## Supplementary Material

Additional file 1 Plants reported to be used by Brou, Saek and Kry during pregnancy, childbirth and postpartum healthcare. Click here for file
