# Supplementary material for: Plants used during pregnancy, childbirth and postpartum healthcare in Lao PDR: A comparative study of the Brou, Saek and Kry ethnic groups
Source: J Ethnobiol Ethnomed. 2009 Sep 8;5:25. doi: 10.1186/1746-4269-5-25 (PMC2749814; doi:10.1186/1746-4269-5-25)
Supplement: Additional file 1 — Plants reported to be used by Brou, Saek and Kry during pregnancy, childbirth and postpartum healthcare. [file 1746-4269-5-25-S1.pdf]

Scientific name	Local name[a]	Voucher[b]	Preparation	Type of Use	Medical use	Part used	Village[c]	Ethnic[d]
Ageratum conyzoides L. (Asteraceae)	Nga Kiou (L)	Kool 501; 518; 634; 653	Leaves used for steambath and washing	Steambath / Body wash	Postpartum recovery	Leaves	8	S
Allium cepa L. (Alliaceae)	Pak Boua (L)	Cultivated	Boiled as vegetable	Boiled	Postpartum recovery	Plant	9	S
Alpinia galanga (L.) Sw. (Zingiberaceae)	Kga (B,S)	Cultivated	Roasted rhizome eaten	Eat	Postpartum recovery	Rhizome	3,8,10	B,S
Alpinia spp. (Zingiberaceae)	Kga (L,S); Pa Proung (B)	Kool 504; 580; ELLA 2; 5; 10	Steambath and washing for recovery[3,4,8,9,10]; Roasting[7]; Roasted rhizome eaten[8,10]	Steambath / Body wash[3,4,8,9,10]; Roasting[7]; Eat[8,10]	Postpartum recovery	Rhizome[1,4,8,9,10]; Stem[1]; Leaves[1,3,7,8,10]	1,3,4,7,8,9,10	B,S
Amomum cf. *microcarpum* C.F. Liang & D. Fang (Zingiberaceae)	Mak Neng Kham (L); Same (B)	Kool 503; HVL 034; ELLA 13; 15; 16; 17	Used for steambath and washing	Steambath / Body wash	Postpartum recovery	Rhizome[3,4,6] Stem[1,2,3,4,6] Leaves[1,2,8]	1,2,3,4,6,8	B,K,S
Amomum spp. (Zingiberaceae)	Mak Neng Khao	Not collected	Rhizome used for steambath and washing	Steambath / Body wash	Postpartum recovery	Rhizome	1,3	B
Artocarpus heterophyllus Lam. (Moraceae)	Mak Mie (L)	Kool 472; ELLA 50	Leaves used for steambath and washing[3]; Peduncle used for more milk[9]	Steambath / Body wash[3]; Decoction[9]	Postpartum recovery[3]; Lactagogue[9]	Leaves[3]; Peduncle[9]	3,9	B,S
Bambusa bambos (L.) Voss (Poaceae)	Pai Paa (L); Pai Tuan (S)	Kool 588	Leaves used for steambath and washing	Steambath / Body wash	Postpartum recovery	Leaves	3,8	B,S
Bambusa blumeana Schult. & Schult. f. (Poaceae)	Pai Baan (L)	Kool 587	Leaves used for steambath and washing	Steambath / Body wash	Postpartum recovery	Leaves	3	B
Bambusa tulda Roxb. (Poaceae)	Mai Bong	Not collected	Leaves used for steambath and washing	Steambath / Body wash	Postpartum recovery	Leaves	3	B
Bischofia javanica Blume (Euphorbiaceae)	Som Fat, Boun Fart (L); Ja Lang Deum (K)	Not collected	Massage breasts with young leaves	External	Lactagogue	Leaves	4,6	B,K

Blumea balsamifera (L.) DC. (Asteraceae)	Bai Naad (L); A Phuak (B); Bu Naek (S)	Kool 614; ELLA 49; 57; 78	Cover bed during roasting for 10-15 days[1,2,9,10]; Place leaves on coals under bed[4,8]	External[1,2,9,10]; Roasting[4,8]	Postpartum recovery	Leaves	1,2,4,8,9,10	B,S
Brassica juncea (L.) Czern. (Brassicaceae)	Pak Khaad (L)	Cultivated	Boiled as vegetable, against headache and to improve milk	Boiled	Postpartum recovery; Lactagogue	Plant	7,8	S
Brassica oleracea L. (Brassicaceae)	Pak Ka Lam (L)	Cultivated	Boiled as vegetable	Boiled	Postpartum recovery	Plant	7	S
Calamus rudentum Thwaites (Arecaceae)	Boun Wai (L); Njod Woun (S); Pull (K)	Kool 668	Shoots boiled as vegetable (S, K); or grilled (K)	Boiled	Postpartum recovery	Shoots	6,8,9,10	K,S
Camellia sinensis (L.) Kuntze (Theaceae)	Bai Chai (L)	Cultivated	Infusion drunk 10 days following parturition	Infusion	Postpartum recovery	Leaves	3	B
Centella asiatica (L.) Urb. (Araliaceae)	Pak Nok (L)	Kool 523; 553	Treatment of hot fever in babies up to 6 months. Place a handful in a bowl of cold water for 5-30 min, then drink and/or wash the infant	External; Cold extract	Infant care	Plant	7	S
Chromolaena odorata (L.) R. M. King & H. Rob. (Asteraceae)	Nga Yerraman (L)	Kool 540; 632; 648	Place young leaves on coals[4]; Cover bed during roasting[1]	Roasting	Postpartum recovery	Leaves	1,4	B
Claoxylon indicum (Reinw. ex Blume) Hassk. (Euphorbiaceae)	Kok Krasell (L); Koh Krasèll (S)	Kool 621; 797	Leaves used for decoction	Steambath / Body wash	Postpartum recovery	Leaves	9,10	S
Cyathula prostrata (L.) Blume (Amaranthaceae)	Tong Chod (L)	Not collected	Plant used for steambath and washing	Steambath / Body wash	Postpartum recovery	Plant	6	K
Dioscorea sp. nov. (Dioscoreaceae)	Tha Thaet Tai; Taet Tae	Kool 645	To treat fever in children. Root used as decoction to wash the infant	Decoction	Infant care	Root	9	S
Elaeagnus conferta Roxb. (Elaeagnaceae)	Kheua Mak Rod (L)	Kool 589	To have enough milk if you are breastfeeding several children at the same time	Decoction	Lactagogue	Leaves; Stem	8	S

Unidentified (Euphorbiaceae)	Khamborsouk (B, S)	Kool 469	Leaves used for steambath and washing[3a]; Boil a handful of roots and leaves in a pot of water until the water level has gone down 2 fingers[3b]; Remove bark through burning and use wood. Not during roasting[10]	Steambath / Body wash[3a]; Decoction[3b,10]	Postpartum recovery	Leaves[3a,b]; Roots[3b]; Wood[10]	3,10	B,S
Unidentified (Euphorbiaceae)	Thadrok Ngoua (S)	Kool 557	Remove bark through burning and use wood. Not during roasting	Decoction	Postpartum recovery	Wood	10	S
Eurycoma longifolia Jack (Simaroubaceae)	Kok Yik Bor Thong (L)	Kool 595	To treat rash in infants. Leaves used for decoction used for washing	External	Infant care	Leaves	9	S
Ficus hispida L.f. (Moraceae)	Doua Pong (L); Je Lang Ka Tod (K)	Kool 511	Leaves used for steambath and washing	Steambath / Body wash	Postpartum recovery	Leaves	6	K
Gigantochloa parvifolia (Brandis ex Gamble) T.Q. Nguyen (Poaceae)	Mai Sod (L); Pho Tioy (B); Mai Or (S); Balre Pa Choy (K)	Kool 471	Leaves used for steambath and washing[3,6]; Shoots boiled as vegetable[9,10]	Steambath / Body wash[3,6]; Boiled[9,10]	Postpartum recovery	Leaves[3,6]; Shoots[9,10]	3,6,9,10	B,K,S
Globba spp. (Zingiberaceae)	Waen (L); Ngaal (S)	Kool 590	Rhizome boiled alone or together with Mai Puai. Decoction drunk 2-3 times daily for 2 months to get good milk	Decoction	Postpartum recovery; Lactagogue	Rhizome	8,9	S
Gonocaryum lobbianum (Miers) Kurz (Icacinaceae)	Seng Muang (L)	Kool 470	Leaves used for steambath and washing[1,3]; Massage breasts with young leaves[6]	Steambath / Body wash[1,3]; External[6]	Postpartum recovery[1,3]; Lactagogue[6]	Leaves	1,3,6	B,K
Hedychium sp. (Zingiberaceae)	Saay Houn (L); Je Lang Yaal (K)	Kool 506	Root eaten as vegetable after birth	Boiled	Postpartum recovery	Roots	6	K
Imperata cylindrica (L.) P. Beauv. (Poaceae)	Nga Kha (L,B)	Kool 483	Entire plant used for steambath and washing	Steambath / Body wash	Postpartum recovery	Plant	3,6	B,K
Ipomoea aquatica Forssk. (Convolvulaceae)	Pak Boung (L)	Cultivated	Boiled as vegetable	Boiled	Postpartum recovery	Plant	7	S

Knema sp. (Myristicaceae)	Ka Sec R'Jae (K); Kheua Leuad (L)	Kool 500	Infusion of the roots is drunk from 2 days up to 2 months after the delivery	Decoction	Postpartum recovery	Roots	5,6	K
Lagerstroemia calyculata Kurz; *L. macrocarpa* Kurz (Lythraceae)	Mai Puai (L); Koh Raan, Koublau (S); Kun Siliang (B)	Kool 515; 547; 679; 837	Decoction of bark drunk during confinement[4,9,10]; Take about 30 cm of stem, roast it clean, chop finely, boil, and drink for 1 month postpartum[7,8]	Decoction	Postpartum recovery	Bark[4,9,10]; Wood[7,8]	4,7,8,9,10	B,S
Lygodium salicifolium C. Presl (Lygodiaceae)	Pak Kot Kheua (L); Ni Ray Koy (B)	ELLA 26	Prevents sickness and dizziness when leaving the bed after the roasting period	Steambath / Body wash	Postpartum recovery	Plant	2	B
Maesa sp. (Myrsinaceae)	Kok Mak Deuan (L); Ko Mak Kok (S)	Kool 543; 842	Boiled roasted wood is drunk	Decoction	Lactagogue	Wood	10	S
Mangifera indica L. (Anacardiaceae)	Mak Muang (L)	Cultivated	Leaves used for steambath and washing	Steambath / Body wash	Postpartum recovery	Leaves	3	B
Melicope viticina (Wall. ex Kurtz) T.G. Hartley (Rutaceae)	Kok Maad (L); Koh Maad (S)	Kool 551; 570; 598	Drink decoction of the leaves[4,9,10]; Use leaves for roasting[7,8,10]; Put leaves on bed and rest[10]	Steambath / Body wash[4,9,10]; Roasting[7,8,10]; External[10]	Postpartum recovery	Leaves	4,7,8,9,10	B,S
Musa acuminata Colla (Musaceae)	Mak Pie Kuay (L); Eupong (B); Mak Pri (S); Ta Lou Ma La (K)	Not collected	Juvenile inflorescence boiled as vegetable	Boiled	Postpartum recovery; Lactagogue	Flower	4,5,6,7,8,9,10	B,K,S
Ocimum basilicum L. (Lamiaceae)	Ee Toh Thai (L)	Cultivated	Anthelminthic for infants: Use one handful of leaves and stems. Put in cold water and drink.	Cold extract	Infant care	Leaves	7	S
Persea odoratissima (Nees) Kosterm. (Lauraceae)	Kok Mai Hoom (L)	ELLA 21	Leaves and singed stem for steambath; massage breasts with young leaves	Steambath / Body wash; External	Lactagogue	Leaves; Stem	2	B

Phoebe lanceolata (Nees) Nees (Lauraceae)	Paivenh (L); Kok Yuang; Kok Tan Tae (S)	Kool 538; 619	Leaves used for steambath and washing[2,3,7]; Roasting[9]	Steambath / Body wash[2,3,7]; Roasting[9]	Postpartum recovery	Leaves	2,3,7,9	B,S
Piper lolot C. DC. (Piperaceae)	Pak Ieleud (L); Prak Leud (S)	Kool 478	Boiled as vegetable[8a,9,10]; coalescence[8b]; To treat hot fever in infants: Apply poultice to the head. Start when the child gets sick and continue up to 3-4 months[7]	Steambath / Body wash[8]; Boiled[8,9,10]; Poultice[7]	Postpartum recovery[8,9,10]; Infant care[7]	Leaves	7,8,9,10	S
Polyalthia cerasoides (Roxb.) Bedd. (Annonaceae)	(Nam) Tao Noi (L)	Kool 513; 575	Leaves used for roasting[3]; Drink a decoction of the wood[2,6]	Roasting[3]; Decoction[2,6]	Postpartum recovery[3,6]; Recover after miscarriage[2]	Leaves[3]; Wood[2,6]	2,3,6	B,K
Psychotria sarmentosa Blume (Rubiaceae)	Van Chod (L); Ko Brao (S); Sala Ba La (K)	Kool 468; ELLA 3; 22; 163	Drink a decoction of the wood for up to 2 months	Decoction	Postpartum recovery[1,2,4,6,7,9,10]; Lactagogue[1]	Wood	1,2,4,6,7,9,10	B,K,S
Rhapis laosensis Becc. (Arecaceae)	Kok Sarn (L)	Kool 571	Decoction of the root	Decoction	Recover after miscarriage	Roots	2	B
Rubus tonkinensis F. Bolle, *R. cochinchinensis* Tratt. (Rosaceae)	Katoum Deng (L)	Kool 542; 552; 574; 631	Infusion	Infusion	Menstruation problems	Leaves	9	S
Sida retusa L. (Malvaceae)	Nga Khad (B)	Kool 474	Entire plant used for steambath and washing	Steambath / Body wash	Postpartum recovery	Plant	3	B
Smilax spp. (Smilacaceae)	Kheua Ya Hua (L); Tang (K)	Kool 550; 774; 782; 824	Leaves used for steambath and washing[5]; First day after delivery: remove bark from stem, chop finely, boil and drink 2-3 times[6]	Steambath / Body wash[5]; Decoction[6]	Postpartum recovery	Leaves[5]; Stem[6]	5,6	K
Tacca chantrieri André (Taccaceae)	Kok Tui (L); Touis (K)	Kool 505	Boiled root eaten postpartum. Bitter	Boiled	Postpartum recovery	Roots	5,6	K

Zingiber officinale Roscoe (Zingiberaceae)	Khing (L); Ai Seuj (B)	Cultivated	Crush rhizome and put in the ear	External	Postpartum recovery (Postpartum haemorrhage)	Rhizome	4	B
Unidentified (Zingiberaceae)	Koh Pa Kang (L); Ta Kong Kalua (B)	ELLA 18	Stops secondary postpartum haemorrhage	Decoction	Postpartum recovery (Postpartum haemorrhage)	Rhizome	2	B
Unidentified (Zingiberaceae)	Pi Din (L)	ELLA 4	Stem and leaves used for steambath and washing	Steambath / Body wash	Lactagogue	Stem; Leaves	2	B
Ziziphus attopoensis Pierre (Rhamnaceae)	Kham Lang seghon (L); Tha Sua Ghon (S)	Kool 546	Remove bark through burning and use wood. Not during roasting	Decoction	Postpartum recovery	Wood	10	S
Ziziphus oenophila (L.) Mill. (Rhamnaceae)	Kok Lep Meo (L); Si Mu Chong (B)	Kool 680	Alleviate postpartum abdominal pain; Expel the placenta	Decoction	Postpartum recovery; Parturition	Wood	2	B

[a]Languages are abbreviated: Lao (L), Brou (B), Saek (S) and Kry (K). [b]Vouchers of all collections are deposited at the Uppsala University Herbarium (UPS) and the herbarium of the Biology Department, National University of Laos; [c]Villages are numbered: 1. Ka Oy; 2. Mak Feuang; 3. Phung; 4. Koutnae; 5. Maka Tai; 6. Maka Kang; 7. Dteun; 8. Buuk; 9. Nameo; 10. Nameuy; [d]Ethnic groups are abbreviated: Brou (B), villages 1-4; Kry (K), villages 5-6; Saek (S), villages 7-10.
